# Healing the Open Apex: A Case Report on Innovative Apexogenesis of a Maxillary Molar Using Bio-C Repair

**DOI:** 10.7759/cureus.76566

**Published:** 2024-12-29

**Authors:** Ashwija Shetty, Hajira A Sultana, Keerthan B V, Nithin S Reddy

**Affiliations:** 1 Department of Conservative Dentistry and Endodontics, The Oxford Dental College, Bengaluru, IND

**Keywords:** apexogenesis, bioceramic cement, bio- c repair, molar, open apex, pulpotomy

## Abstract

Preserving pulp vitality in developing permanent teeth is paramount. This approach facilitates continued root formation, ultimately leading to apical closure, enhanced root strength, and improved overall tooth integrity. This case report details the management of a 17-year-old female patient presenting with dental caries on the right permanent maxillary molar. Clinical and radiographic examinations revealed reversible pulpitis in the tooth with an immature apex. The case highlights the use of a novel bioceramic cement, Bio-C Repair (Angelus, Londrina, Brazil), for apexogenesis. Treatment was performed under rubber dam isolation. This involved caries removal and extirpation of the inflamed coronal pulp. Subsequently, the residual radicular pulp was covered with Bio-C Repair cement. Follow-up examinations at one, three, and six months demonstrated normal tooth function and a normal response to pulp sensibility tests. No signs or symptoms of pulpal pathology were observed. The radiographic evaluation confirmed complete root development. Therefore, Bio-C Repair, with its convenient ready-to-use format, can facilitate faster and more efficient management of open apices.

## Introduction

Dental caries poses a significant threat during the development of teeth. Consequently, it can lead to irreversible damage to the dental pulp, ultimately resulting in pulpal necrosis and arrested root development. Preserving pulp vitality should be the primary objective in the treatment of immature permanent teeth [1]. This facilitates continuous root development, resulting in apical closure, enhanced root strength, and improved structural integrity [2]. Apexogenesis is defined as a vital pulp therapy technique for immature teeth, promoting continued root formation and subsequent apical closure [3].

Several materials can be employed for apexogenesis, including calcium hydroxide, mineral trioxide aggregate (MTA), calcium-enriched mixture (CEM), biodentine, portland cement, and enamel matrix derivative [4].

Bio-C Repair (Angelus, Londrina, Brazil), a recently introduced novel calcium silicate-based endodontic cement, demonstrates bioactivity, fostering tissue repair and biomineralization. This material exhibits several advantages, such as diminished moisture sensitivity compared to conventional alternatives and streamlined application through a convenient single-use syringe format, thereby enhancing clinical efficiency [5].

This case report illustrates the successful use of Bio-C Repair, a newly introduced bioceramic material, in achieving apexogenesis of an immature permanent maxillary molar exhibiting reversible pulpitis.

## Case presentation

**Figure 1:**
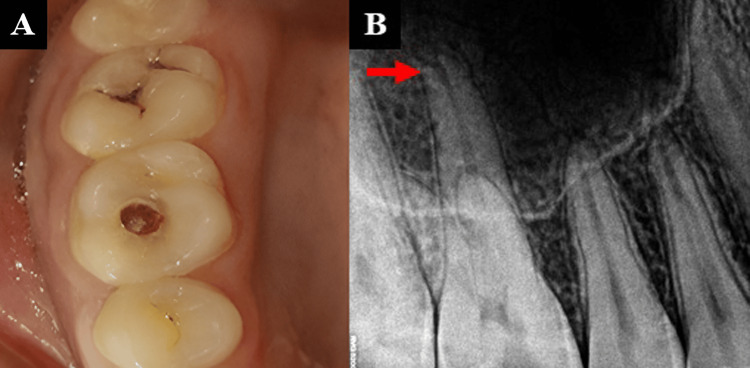
(A) Pre-operative clinical image and (B) pre-operative radiograph revealing an open apex.

A 17-year-old female patient presented to the department reporting pain in her upper right back tooth region for the past 15 days. The pain was sudden in onset, sharp, and intermittent, with moderate intensity. Clinical examination revealed deep dentinal caries in the maxillary right first permanent molar (Figure 1A). Pulp sensibility testing with cold test (Roeko, Endo-Frost, Coltene/Whaledent, Langenau, Germany) demonstrated an exaggerated response. The radiographic assessment identified deep dental caries involving the pulp chamber with an immature palatal root (Figure 1B).

Clinically and radiographically, a diagnosis of symptomatic reversible pulpitis with an immature apex was established. Following informed consent from both the patient and the family, vital pulp therapy was elected as the treatment plan.

**Figure 2:**
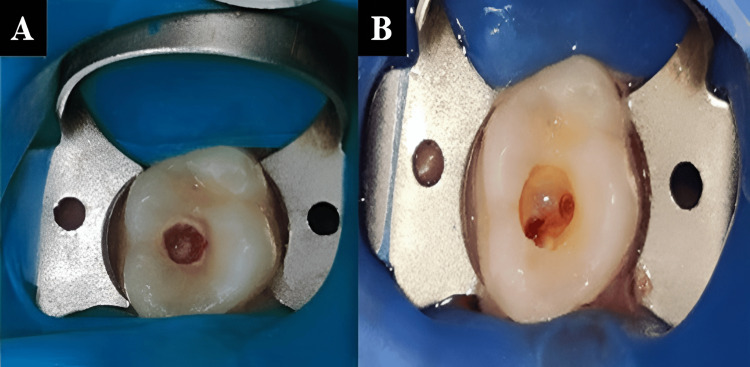
(A) Rubber dam isolation of the tooth and (B) pulpal exposure following removal of carious lesion.

Local anesthesia was administered with local infiltration of 1.8 mL of 2% lignocaine and epinephrine (1:80,000), followed by rubber dam isolation (Figure 2A). The carious lesion was extirpated with a sterile #3 round bur and high-speed handpiece (NSK Pana Air FX, Nakanishi Inc., Tochigi, Japan) with water coolant (Figure 2B).

**Figure 3:**
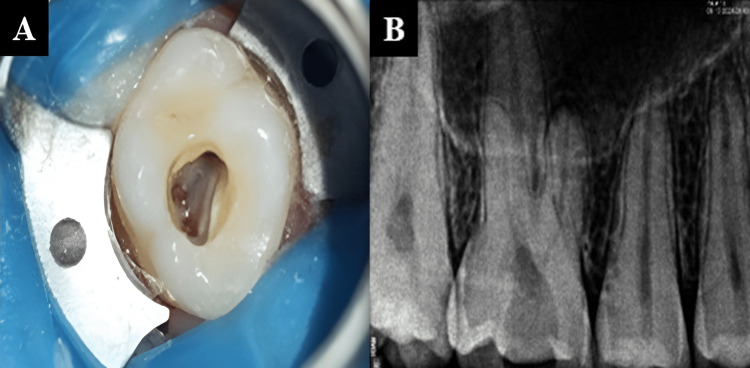
(A) Pulp extirpation under dental operating microscope and (B) radiograph after pulp extirpation.

The coronal pulp was exposed and extirpated under a dental operating microscope at 8× magnification (Figures 3A-3B).

The cavity was irrigated with 5% sodium hypochlorite (NaOCl) solution for 30 seconds, and the exposed pulp was evaluated. The pulp exhibited a healthy, bright red appearance. Hemostasis was established by exerting gentle pressure with a cotton pellet impregnated with 5% NaOCl solution for four minutes.

**Figure 4:**
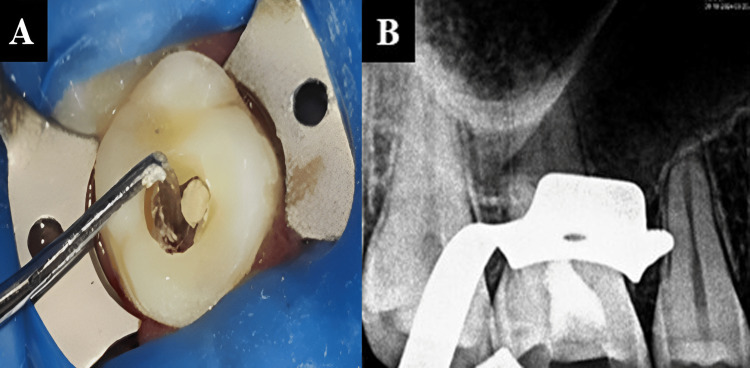
(A) Placement of Bio-C Repair into pulp chamber and (B) radiograph taken after placement of Bio-C repair.

Bio-C Repair was then introduced into the pulp chamber to an approximate thickness of 3 to 4 mm (Figures 4A-4B).

**Figure 5:**
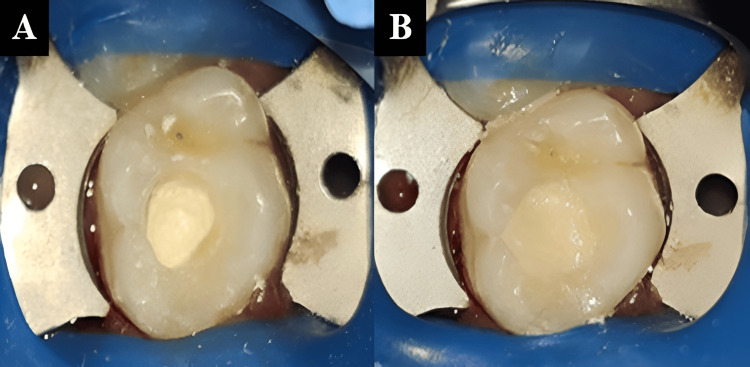
(A) Placement of glass ionomer cement and (B) placement of composite restoration.

This was followed by the placement of glass ionomer cement, over which a composite restoration was then placed (Figures 5A-5B). A post-operative radiograph was obtained.

The patient was advised to contact the clinic promptly in the event of any pain or discomfort. The tooth underwent clinical and radiographic assessment at the following intervals: at two weeks and at one, three, and six months.

**Figure 6:**
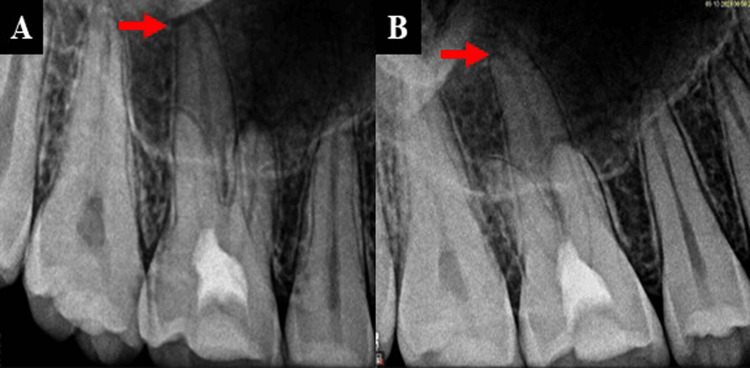
(A) Three-month follow-up radiograph and (B) six-month follow-up radiograph.

The three-month follow-up radiograph demonstrated periapical healing with no signs of periapical pathologies (Figure 6A). At the six-month follow-up, the radiograph demonstrated continued root formation with no signs of pulpal pathology (Figure 6B). Pulp sensibility testing during both visits revealed a normal response.

## Discussion

The primary objective of vital pulp therapy in immature teeth with open apices is to maintain pulp vitality and promote apexogenesis, particularly when these teeth are affected by trauma or deep caries. According to the American Academy of Pediatric Dentistry, pulp capping, partial pulpotomy, and conventional pulpotomy are treatment options for achieving apexogenesis in immature teeth with open apices [6].

The selection between partial and complete pulpotomy is determined by the extent of inflamed and infected pulp tissue and the amount of remaining healthy pulp. In cases of extensive carious lesions, bacterial contamination of the coronal pulp is likely. Therefore, mechanical removal of a portion of the infected pulp tissue is generally preferred over direct pulp capping. This may explain the higher success rates reported for complete pulpotomy, as it involves the removal of a larger volume of potentially infected pulpal tissue compared to partial pulpotomy [7,8]. Hence, complete pulpotomy was considered.

Pulp sensibility testing and clinical presentation alone are insufficient to definitively differentiate between reversible and irreversible pulpitis [9].

Clinical diagnosis, particularly the color and bleeding time observed during pulp exposure, can provide valuable insights into the extent of pulp inflammation. Bright red hemorrhage with a cessation time of less than five minutes is indicative of reversible pulpitis, and profuse cherry red hemorrhage or a fibrous white appearance of the pulp may suggest irreversible pulpitis [10].

Several studies have corroborated the correlation between the extent of pulpal hemorrhage and the intensity of pulp inflammation. Profuse hemorrhage that is hard to control often indicates severe inflammation. Furthermore, if bleeding persists beyond five to 10 minutes, it may suggest severe pulp inflammation, necessitating pulpectomy [11].

Studies have recommended employing magnification techniques to enhance the management of exposed pulp tissue and improve treatment outcomes. The dental operating microscope provides a highly detailed view, enabling a more precise assessment of injured tissues, inspection of the pulp following extirpation, and evaluation of the hemostasis time [12].

In the present case, subsequent to pulpotomy, the pulp stump exhibited bright red bleeding, which was controlled within four minutes.

NaOCl (5%) serves as an antibacterial agent, disinfecting the dentin-pulp interface and removing biofilm, blood clots, and debris, including dentinal chips and damaged cells, at the site of mechanical exposure while preserving pulp vitality. Additionally, NaOCl provides hemostasis, which is crucial for successful pulpotomy procedures [13].

Bio-C Repair is an immediately usable cement demonstrating comparable biocompatibility, bioactivity, and biomineralization properties to conventional materials such as MTA and biodentine. Notably, it exhibits several advantages, including reduced moisture sensitivity, exceptional insolubility, and favorable tissue healing properties. Its convenient single-use syringe format simplifies application and enhances clinical efficiency by reducing procedural time [14]. Furthermore, studies have shown that Bio-C Repair, similar to biodentine, can promote cell viability and odontogenic mineralization [15]. Based on these favorable characteristics, Bio-C Repair was selected for this case.

## Conclusions

Successful outcomes in vital pulp therapy are contingent upon a combination of optimal treatment protocols and the utilization of ideal materials. Bio-C Repair, a novel bioceramic material, can facilitate and accelerate the management of endodontic complications in its ready-to-use format, simplifying procedures and saving time.

Further long-term observational studies are recommended to expand the existing evidence base demonstrating the successful clinical application of this repair cement.
